# Histone deacetylase 1 expression is inversely correlated with age in the short-lived fish *Nothobranchius furzeri*

**DOI:** 10.1007/s00418-018-1687-4

**Published:** 2018-06-28

**Authors:** Gordin Zupkovitz, Sabine Lagger, David Martin, Marianne Steiner, Astrid Hagelkruys, Christian Seiser, Christian Schöfer, Oliver Pusch

**Affiliations:** 1 Center of Anatomy and Cell Biology, Medical University of Vienna, Schwarzspanierstr. 17, 1090 Vienna, Austria; 2 Unit of Laboratory Animal Pathology, University of Veterinary Medicine, 1210 Vienna, Austria; 3 Institute of Molecular Biotechnology of the Austrian Academy of Sciences (IMBA), 1030 Vienna, Austria

**Keywords:** Histone deacetylase, *Nothobranchius furzeri*, Epigenetics, Aging

## Abstract

Aging is associated with profound changes in the epigenome, resulting in alterations of gene expression, epigenetic landscape, and genome architecture. Class I Histone deacetylases (HDACs), consisting of HDAC1, HDAC2, HDAC3, and HDAC8, play a major role in epigenetic regulation of chromatin structure and transcriptional control, and have been implicated as key players in the pathogenesis of age-dependent diseases and disorders affecting health and longevity. Here, we report the identification of class I Hdac orthologs and their detailed spatio-temporal expression profile in the short-lived fish *Nothobranchius furzeri* from the onset of embryogenesis until old age covering the entire lifespan of the organism. Database search of the recently annotated *N. furzeri* genomes retrieved four distinct genes: two copies of *hdac1* and one copy of each *hdac3* and *hdac8*. However, no *hdac2* ortholog could be identified. Phylogenetic analysis grouped the individual killifish class I Hdacs within the well-defined terminal clades. We find that upon aging, Hdac1 is significantly down-regulated in muscle, liver, and brain, and this age-dependent down-regulation in brain clearly correlates with increased mRNA levels of the cyclin-dependent kinase inhibitor *cdkn1a* (*p21*). Furthermore, this apparent reduction of class I HDACs in transcript and protein levels is mirrored in the mouse brain, highlighting an evolutionarily conserved role of class I HDACs during normal development and in the aging process.

**Electronic supplementary material:**

The online version of this article (10.1007/s00418-018-1687-4) contains supplementary material, which is available to authorized users.

## Introduction

In the last years, epigenetic mechanisms have been increasingly recognized as key players during health and disease. Epigenetic changes influencing gene expression throughout the lifespan involve alterations in DNA methylation patterns, posttranslational histone modifications, and chromatin remodeling. The most studied histone modification, acetylation, is reversibly regulated by histone acetyltransferases (HATs) and histone deacetylases (HDACs). HDACs catalyze the removal of acetyl groups from the *N*-ε-acetyl-l-lysine residues in histones and non-histone proteins. As a result of deacetylation, chromatin is susceptible to switch from an open transcriptionally active to a compact transcriptionally inactive state.

So far, 18 different HDACs have been identified. Based on homology to their yeast counterparts, HDACs are classified into four distinct groups (Gregoretti et al. 2004). Class I enzymes reveal homology to the transcriptional repressor *RPD3* and comprise HDAC1, HDAC2, HDAC3, and HDAC8. Class II enzymes, HDAC4, HDAC5, HDAC6, HDAC7, HDAC9, and HDAC10, show homology to *HDA1*-like proteins and participate in defined tissue-specific processes. Interestingly, several class II members shuttle between cytoplasm and nucleus and as they do not contain a catalytically active HDAC domain, rely on the HDAC activity of class I member HDAC3 (HDAC4, HDAC5, and HDAC7). HDAC11 represents the sole member of class IV HDAC enzymes. Sirtuins compose class III HDACs. The founding member of the sirtuin family, yeast *SIR2*, was the first evolutionary conserved gene to be identified as a regulator of longevity (Kaeberlein et al. 1999). In mammals, the sirtuin family comprises seven proteins (SIRT1–SIRT7), which vary in tissue specificity, subcellular localization, enzymatic activity, and targets. Sirtuins constitute a protein family of metabolic sensors, translating changes in NAD+ levels into adaptive responses, thereby acting as crucial regulators of the network that controls energy homeostasis and as such determines health span.

In yeast and fruit flies, *RPD3* was identified as a gerontogene. Deletion of *RPD3* in *S. cerevisiae* results in an increased replicative lifespan (Kim et al. 1999). In Drosophila, systemic down-regulation extends lifespan in *rpd3*−/+ heterozygous mutants, although *rpd3*−/− homozygotes are lethal (Rogina et al. 2002). Similarly, pharmacological inhibition of HDAC activity by feeding fruit flies sodium 4-phenylbutyrate (PBA) shows significant life-extending effects (Kang et al. 2002). Accordingly, HDAC inhibitors and drugs specifically targeting epigenetic pathways have been proposed as promising therapeutics to combat aging (Pasyukova and Vaiserman 2017).

In the present study, we provide a detailed spatio-temporal expression profile of class I HDACs in the emerging aging model *Nothobranchius furzeri*. The turquoise killifish is currently the shortest-lived vertebrate that can be bred in captivity (Genade et al. 2005). Importantly, the short lifespan is associated with typical age-dependent phenotypes and pathologies such as decline in fertility, sarcopenia, cognitive decline, and cancerous lesions. Furthermore, killifish displays age-related telomere shortening (Hartmann et al. 2009) and regression of tissue regeneration potential (Wendler et al. 2015; Hoppe et al. 2015). *N. furzeri* is also responsive to environmental stimuli that affect aging in other species, such as dietary restriction (Terzibasi et al. 2009), a resveratrol-rich diet (Valenzano and Cellerino 2006), and temperature (Valenzano et al. 2006). These characteristics, combined with the recently annotated genome and the toolbox for precise genome-editing make this fish an attractive model organism, uniquely fit to study vertebrate aging, physiology, and age-dependent diseases throughout organismal lifespan (Kim et al. 2016).

Here, we show that Hdac1 is significantly down-regulated in muscle, liver, and brain tissue during aging. This age-dependent down-regulation in brain clearly correlates with increased mRNA levels of the cyclin-dependent kinase inhibitor *cdkn1a* (*p21*). We subsequently aimed to relate these findings to other vertebrate model systems. Collectively, we also observe decreased class I HDAC expression in the aging mouse brain hinting towards an evolutionary conserved mechanism of these epigenetic modifiers in regulating gene expression during the aging process. Due to the potential reversibility of epigenetic changes that occur as a hallmark of aging, a detailed expression atlas of epigenetic modifiers should help to develop chromatin-based strategies for delaying or reversing age-related diseases.

## Materials and methods

### Ethical standards

All applicable international, national, and/or institutional guidelines for the care and use of animals were followed. Animal experiments involving mouse were reviewed and approved by the Austrian ministry authorities (Approval no. BMWF-66.009/0281-I/3b/2012) and conducted according to relevant regulatory standards. All killifish experiments and procedures were in accordance with the ethical standards of the Ethics Committee of the Medical University of Vienna.

### Mouse care and maintenance

Mice were bred in a mixed genetic background C57BL/6J × 129SV. Animals were sacrificed by cervical dislocation at indicated time points (P0—day of birth, adult—8 weeks, and old—104 weeks). Brains were isolated and flash frozen in liquid nitrogen and stored at − 80 °C until used for RNA or protein extraction.

### Killifish care and maintenance

All animals used in this study were raised in the fish colony at the Medical University, Vienna. All experiments were performed using males of the *N. furzeri* strain MZM 0410 (kindly donated by Alessandro Cellerino, Pisa). Maintenance and breeding conditions were performed as previously described (Genade et al. 2005). Fish were grown in an overflow custom made system, fed twice daily with frozen red blood worms, and maintained at 27 °C on a 12 h light/dark cycle with a fish density of one fish per 2,8 l tank. Selected fish were sacrificed at 5 and 31 weeks of age, unless specified otherwise. Fish were euthanized using tricaine methane sulfonate (MS-222) buffered with sodium bicarbonate to a neutral pH. Whole fish or dissected organs were freshly isolated and further processed for IHC or snap-frozen in liquid nitrogen and stored at − 80 °C for RNA or protein analyses.

### Survival curve

For this analysis, two independent hatches consisting of 41 and 52 (total 93) fish were combined. Mixed-sex groups were set up when the animals had reached 5 weeks of age and showed the first signs of sex dimorphism. The initial animal densities in these trials were 8 animals per 25 l tank. Tanks were checked twice a day, and dead animals were instantly recorded and removed. Based on these data, survival was calculated on a weekly basis. During the trial, we did not adjust population density caused by declining group size. Fish survival was presented as percentage of alive fish at a certain age per total number of fish.

### Sequence analysis

Amino acid sequences for all analyzed species, except for *N. furzeri*, were obtained from ENSEMBL (http://www.ensembl.org). Sequences for *N. furzeri* were acquired from the NFINgb genome browser (http://nfingb.leibniz-fli.de) (Reichwald et al. 2015). We considered the following species: human *(Homo sapiens)*, mouse *(Mus musculus)*, frog *(Xenopus tropicalis)*, zebrafish *(Danio rerio)*, spotted gar *(Lepisosteus oculatus)*, medaka *(Oryzias latipes)*, platyfish *(Xiphophorus maculatus)*, coelacanth *(Latimeria chalumnae)*, and lamprey *(Petromyzon marinus)*. To artificially root the phylogenetic tree, we incorporated a remote HDAC protein from the archaebacteria *Sulfolobus islandicus*, that was used as an outgroup. In a first step, the sequences were compared using the Muscle program to obtain high-quality multiple alignments. Low conservation parts of the alignment were filtered out using the GBlocks software. Phylogenetic tree was generated with the maximum-likelihood algorithm from the PhyML program using the LG substitution matrix. Finally, the corresponding table of sequence similarity was derived from the alignment using Geneious software. List of gene identifiers from which the protein sequences were retrieved is listed in Supplementary Table 1.

### RNA isolation and quantitative real-time RT-PCR analysis

Total RNA was isolated from frozen fish brain, liver, and muscle or mouse brain samples with TRI reagent (Sigma-Aldrich) as recommended by the manufacturer. 1 µg of total RNA was reverse transcribed with the iScript cDNA synthesis kit (Bio-Rad) and real-time RT-PCRs were performed using SensiMix™ mastermix (Bioline).

### Protein isolation and immunoblot analysis

Total protein extracts and western blot analyses from whole fish, fish tissue, and mouse brain frozen samples were performed as previously described (Hagelkruys et al. 2014). The following antibodies were used for protein detection: HDAC1 (ab33278, Abcam), HDAC2 3F3 (Millipore), HDAC3 (ab7030, Abcam), HDAC8 (ab134747, Abcam), and ß-Actin (#4967, CST). Detailed information about antibody specification including producer and working dilution can be found in Supplementary Table 2.

### Immunohistochemistry

Histological and IHC analysis was performed as previously described (Murko et al. 2013). Fluorescence labeling was carried out with Tyramide Signal Amplification Kit (PerkinElmer) according to the manufacturer’s instructions. Detailed information about antibody specification including producer and working dilution can be found in Supplementary Table 2.

### Microscopy

IHC stainings of the sections and whole embryos were imaged and captured on a Nikon Eclipse E800 fluorescence microscope equipped with a Nikon DS-Ri1 camera.

### Statistical analysis

Real-time PCR experiments were evaluated with Microsoft Excel and Prism GraphPad software. The significance between groups was determined by the unpaired Student’s *t* test. *P* values were calculated with the Prism software and standard deviation (sd) is shown. *p < 0.05; **p < 0.01; ***p < 0.001.

## Results

### Phylogenetic analysis reveals high evolutionary conservation of killifish class I Hdacs

**Fig. 1:**
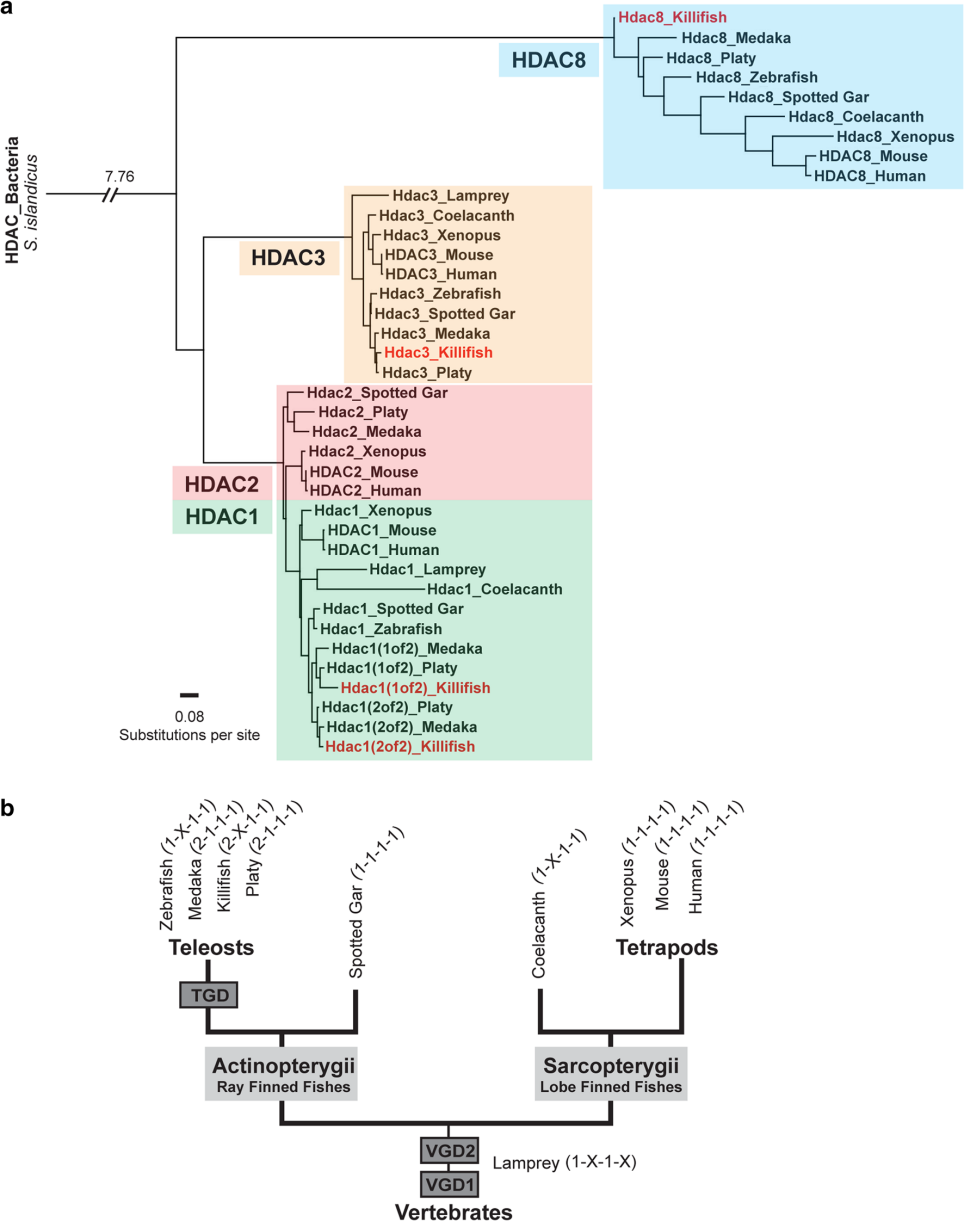
Phylogenetic relationships between selected animal class I HDAC proteins. **a** Phylogenetic tree of class I HDACs based on full-length protein sequences was constructed by the maximum-likelihood algorithm. Remote HDAC protein from the archaebacteria *Sulfolobus islandicus* was used as an outgroup. We considered the following species: human (*Homo sapiens)*, mouse *(Mus musculus*), frog *(Xenopus tropicalis)*, zebrafish *(Danio rerio)*, spotted gar *(Lepisosteus oculatus)*, medaka *(Oryzias latipes)*, platyfish *(Xiphophorus maculatus)*, killifish (*Nothobranchius furzeri)*, coelacanth *(Latimeria chalumnae)*, and lamprey *(Petromyzon marinus)*. **b** Schematic illustrating evolutionary relationships of vertebrates. VGD1 and VGD2 represent the two rounds of the early vertebrate genome duplication that occurred before the divergence of ray-finned and lobe-finned fish. TGD depicts the teleost-specific whole-genome duplication predating the teleost radiation. Spotted gar represents the unduplicated sister group of teleosts. Coelacanth is a sarcopterygian fish illuminating the transition to a terrestrial life, giving rise to modern tetrapods. Lamprey constitutes the most ancient lineage of vertebrates. Copy number of individual class I HDACs is set in parentheses

To identify class I Hdac orthologs in killifish, we analyzed the recently sequenced *N. furzeri* genome (Reichwald et al. 2015; Valenzano et al. 2015). Data mining in the *N. furzeri* Genome Browser (http://nfingb.leibniz-fli.de) retrieved four killifish genes that are highly related to human and mouse class I *HDACs*: 2 copies of *hdac1* (1 of 2: *Nfu_g_1_016470*; 2 of 2: *Nfu_g_1_014999*), and one ortholog of each *hdac3* (*Nfu_g_1_012108*) and *hdac8* (*Nfu_g_1_009605*). Interestingly, no *hdac2* ortholog was identified. To examine the evolutionary relationships of killifish class I Hdacs within the vertebrate genomes, we performed phylogenetic analysis on amino acid sequences of class I HDAC homologs of human, mouse, xenopus, coelacanth, spotted gar, zebrafish, medaka, platy, and killifish, as well as the jawless lamprey, the most ancient lineage of vertebrates describing an intermediate phylogenetic position between invertebrates and gnathostomes. We included coelacanth because of its status as a living fossil, representing sarcopterygian fish which constitutes a sister group of tetrapods. Therefore, coelacanth exemplifies the transition of vertebrates from water to land (Amemiya et al. 2013). In addition, the spotted gar constitutes a ray-finned fish that diverged from teleosts before the teleost-specific genome duplication (Braasch et al. 2016). A phylogenetic tree constructed by the maximum-likelihood method revealed that individual killifish class I Hdacs allocated unambiguously to the well-defined clades, showing highest conservation with other teleost species (Fig. 1a). Within the selected teleosts, Nothobranchius grouped most closely with medaka, which is fully consistent with the taxonomic classification of the analyzed fish species. Pairwise sequence comparison scoring similarity between killifish and human full-length proteins further demonstrates the high degree of class I HDAC conservation: HDAC1 (1 of 2) 93.7%, HDAC1 (2 of 2) 95.1%, HDAC3 98.1%, and HDAC8 88.1% (Supplementary Table 3). Moreover, sequence similarities of individual class I HDACs between species are higher than homologies within the same species. Most vertebrates analyzed harbor one copy of each class I HDAC (Gregoretti et al. 2004), whereas, in fish, a more complex situation is observed (Fig. 1b). For example, in zebrafish, no ortholog of *hdac2* has been identified. Scanning both Nothobranchius genome browsers (NFINgb: http://nfingb.leibniz-fli.de; Stanford Killifish Genome Browser: http://africanturquoisekillifishbrowser.org) as well as the transcriptome browser (NFINtb: http://nfintb.leibniz-fli.de/nfintb/) (Petzold et al. 2013) did not retrieve an *hdac2* ortholog. However, the killifish genome harbors two copies of *hdac1*, a paralog configuration that is also observed in other fish species such as medaka and platy. In addition to 2 copies of *hdac1*, medaka and platy further encode an ortholog of *hdac2*. The spotted gar whose genome represents the unduplicated sister group of teleosts possesses one ortholog of each class I *hdac* resembling the copy number composition of most tetrapods analyzed, including mammals (Gregoretti et al. 2004). Although coelacanth reveals a comparable ortholog configuration to zebrafish, the individual class I *hdacs* grouped consistently with the tetrapod lineage in the phylogenetic tree underlining its status as a living fossil and connecting link during vertebrate HDAC evolution.

### Ubiquitous expression of class I Hdacs in killifish embryos

In general, mammalian class I HDACs are broadly expressed throughout embryonic development but later diversify and give rise to tissue-specific expression patterns in the adult organism (Brunmeir et al. 2009). Recent evidence from expression pattern reports in mouse and chick suggests a spatio-temporal expression profile of class I HDACs in various developmental stages and tissues (Murko et al. 2010). In addition, loss of function studies in mouse hint towards essential functions of class I HDACs in early development, as knockout of HDAC1 and HDAC3 exhibit early embryonic lethality (Lagger et al. 2002; Knutson et al. 2008; Montgomery et al. 2008) and deletion of HDAC2 and HDAC8 lead to peri-natal lethality due to heart defects (Trivedi et al. 2007; Montgomery et al. 2007) or skull malformations (Haberland et al. 2009).

**Fig. 2:**
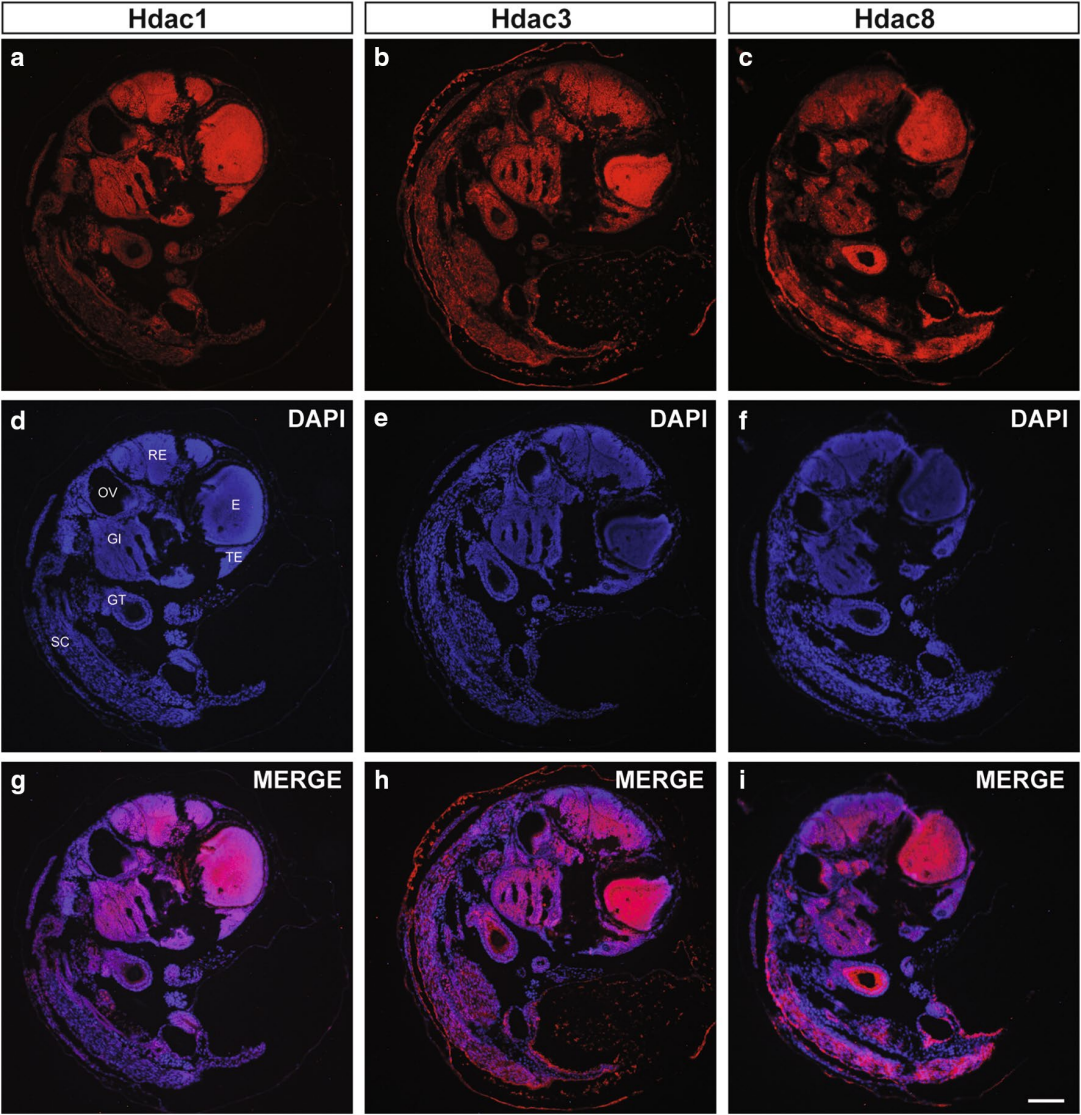
Ubiquitous expression of Class I Hdacs in *N. furzeri* embryos. Fluorescence immunohistochemistry (IHC) stainings of **a** Hdac1, **b** Hdac3, and **c** Hdac8 on sections from 15 day old embryos. Nuclei are counterstained with DAPI (**d–f**) and merged with signals obtained from class I Hdac-specific stainings (**g–i**). *GI* gills, *GT* gut tube, *E* eye, *OV* otic vesicle, *RE* rhombencephalon, *SC* spinal cord, *TE* telencephalon. Scale bar: 100 μm

To determine whether *N. furzeri* class I Hdacs show comparable expression patterns to previously analyzed organisms during embryogenesis, we started our survey by performing fluorescence immunohistochemistry stainings on embryogenesis completed black eye-stage (embryonic day E14) killifish sections. We detected robust and ubiquitous expression of all analyzed class I Hdacs throughout *N. furzeri* embryos (Fig. 2). Hdac1 and Hdac3 particularly overlapped and revealed a pronounced staining in the mesencephalon (ME) region of the brain and the eye (E). We also found prominent Hdac1 and Hdac3 positive staining in the rhombencephalon (RE) and the gills (GI). Hdac8 similarly revealed strong staining in the mes, and rhombencephalon, but in contrast to Hdac1 and Hdac3 further robust staining in the gut tube (GT) and the spinal cord (SC) regions of the embryo. Overall, we found ubiquitous expression of all class I Hdacs in the developing *N. furzeri* embryo but equally detected several tissue-specific expression differences.

### Differential expression levels of class I Hdac members during aging

**Fig. 3:**
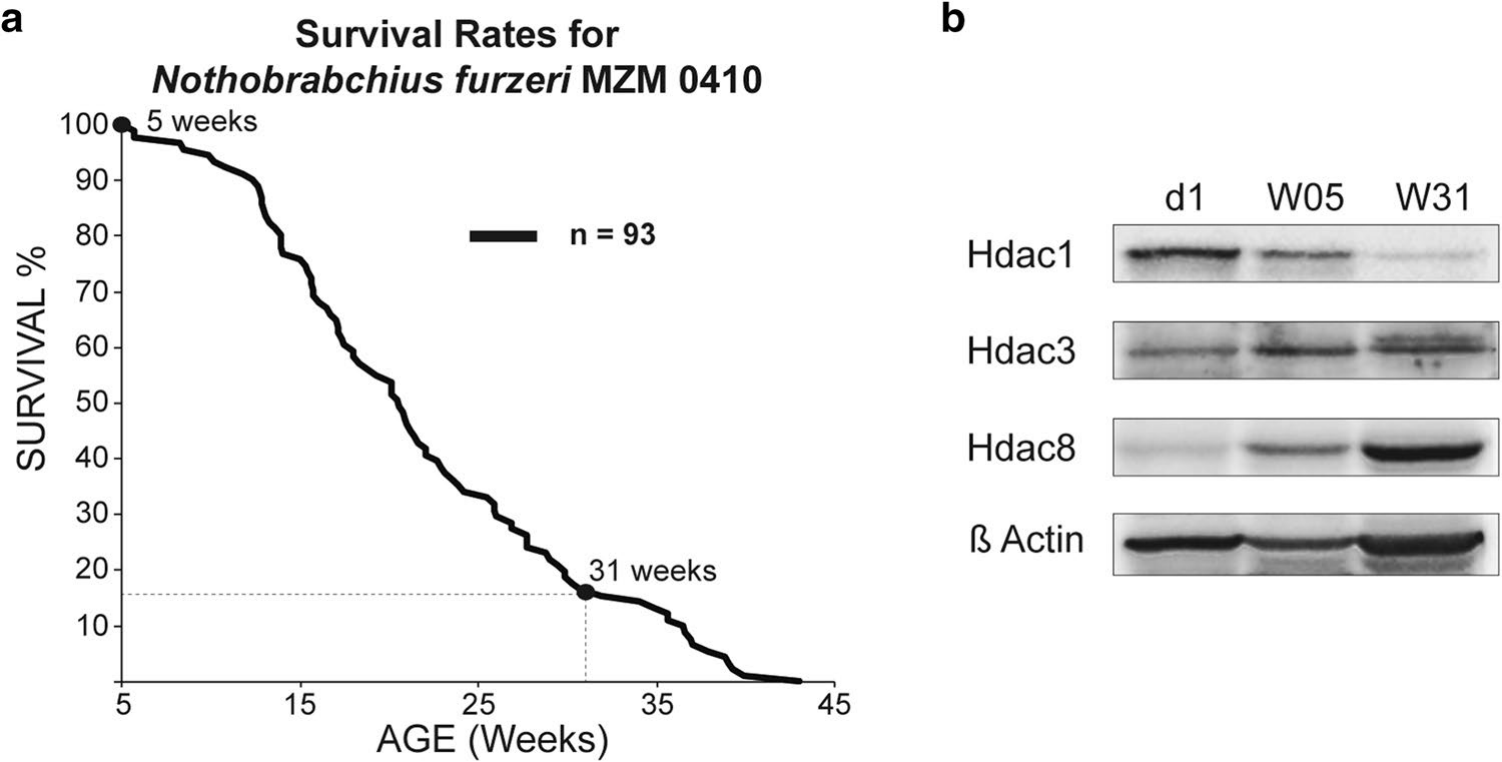
Differential expression levels of class I Hdac members during aging. **a** Age-dependent survivorship of *N. furzeri* strain MZM 0410. Total number of fish was *n* = 93. **b** Western blot of whole fish protein extracts at day of hatching (d1) and 5 (W05) and 31 (W31) weeks of age. Extracts were analyzed for expression of Hdac1, Hdac3, and Hdac8. β-Actin was used as a loading control

Recent experiments in *S. cerevisiae, C. elegans*, and *D. melanogaster* implied a function for various Hdacs in lifespan regulation that could be achieved *via* their control of essential cellular processes, such as oxidative damage, metabolic control, or differential gene expression (Chang and Min 2002). As the use of HDAC inhibitors such as phenylbutyrate has been shown to increase lifespan in these organisms (Pasyukova and Vaiserman 2017), dynamic expression and/or activity of HDACs in the aging process is conceivable and needs to be rigorously studied. To test for possible expression differences of class I Hdacs during the *N. furzeri* life cycle, we analyzed three different time points during the aging process: day of hatching (d1), W05 (5 weeks old), and W31 (31 weeks old). At W05, *N. furzeri* reaches sexual maturity and is considered a young adult. Age W31 roughly corresponds to 15% survivorship in our colony, where individuals show a drastic decline in performance and display most aging-related features. Fish reaching this age are considered old adults (Fig. 3a). Lifespan analysis of the MZM *N. furzeri* strain conducted in our fish facility compares well with reports from other laboratories (Graf et al. 2010). To correlate the survival rate with class I Hdac expression levels, we performed western blotting of whole killifish protein extracts in an age-dependent time course (Fig. 3b). Interestingly, we found that Hdac1 showed a pronounced age-dependent decrease on the protein level, whereas Hdac3 seemed to be stably expressed throughout the individual age groups. Hdac8 showed an inverse temporal regulation compared to Hdac1 as it was found up-regulated upon aging. Our results reveal differential expression levels of class I Hdacs in whole fish extracts during aging hinting towards divergent functions of individual class I members.

### Tissue-specific expression of class I Hdacs

**Fig. 4:**
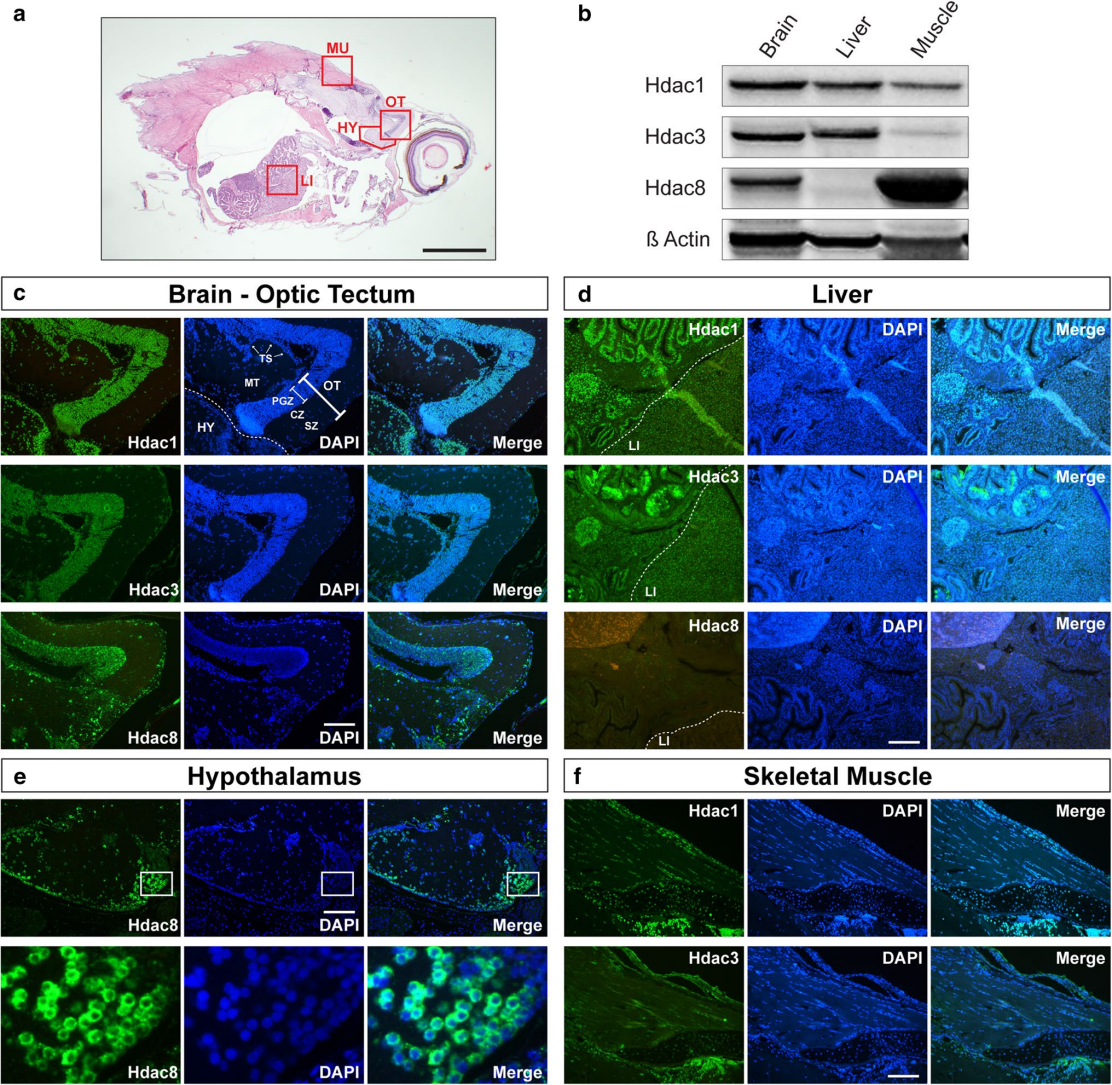
Tissue-specific expression of class I Hdacs. **a** Hematoxylin and eosin staining of a sagittal section of 5 weeks old fish. Red rectangles show regions analyzed in detail by fluorescent IHC. Scale bar is 1 mm. **b** Western blot analysis of total protein extracts obtained from brain, liver, and muscle of 5 weeks old fish. Extracts were analyzed for the expression of Hdac1, Hdac3, and Hdac8. β-Actin was used as a loading control. **c** Fluorescence IHC stainings of Hdac1, Hdac3, and Hdac8 on brain—optic tectum region from 5 weeks old *N. furzeri*. Dashed line presents border between optic tectum and hypothalamus. **d** IHC stainings of Hdac8 on brain—hypothalamic region of 5 weeks old fish. Red rectangle in upper panel shows the zoomed region of hypothalamus presented in lower panel. **e** IHC stainings of Hdac1, Hdac3, and Hdac8 on liver of 5 weeks old fish. Dashed line shows border region between gut, pancreas, and liver. **f** IHC stainings of Hdac1 and Hdac3 on skeletal muscle of 5 weeks old *N. furzeri*. Nuclei were counterstained with DAPI (**c–f** middle panels). *CZ* central zone, *HY* hypothalamus, *LI* liver, *MT* midbrain tegmentum, *MU* muscle, *OT* optic tectum, *PGZ* periventricular grey zone, *SZ* superficial zone, *TS* torus semicircularis. Scale bar: 50 μm

Mouse loss of function studies and expression pattern analysis suggest a tissue-specific function for individual class I HDACs during development. To gain insight into organ-specific expression of class I Hdacs in killifish, we analyzed neuronal, metabolic, and locomotor tissues (Fig. 4a) as these structures are particularly prone to behavioral, structural, or molecular alterations during the aging process. First, we performed western blotting on brain, liver, and skeletal muscle extracts of killifish young adults (week five; Fig. 4b). Hdac1 and Hdac3 were detected in all organs studied, although Hdac3 was expressed at a low level in skeletal muscle tissue. Interestingly, Hdac8 expression differed markedly from the other class I Hdacs, and was robustly expressed in brain and skeletal muscles while absent in liver. To investigate class I Hdac expression in the spatial context and to gain information about the subcellular localization, we next performed immunohistochemistry (IHC) in all three tissue types (Fig. 4c–f). All three Hdacs were strongly expressed in the brain. Particularly high levels of class I Hdac expression were present in midbrain, most evident in the optic tectum (OT) and in the hypothalamic region (HY) (Fig. 4c, e). In the optic tectum, the signal was strongest in the periventricular gray zone (PGZ) where the majority of nuclei of the optic tectum reside. In the overlaying layers, the central and the superficial zones, neuronal nuclei were predominately positive stained, although a few nuclei were devoid of signal. The deeper layers of the midbrain, the midbrain tegmentum including the torus semicircularis (TS) also showed strongly positive stained nuclei as did neurons in the hypothalamic region (Fig. 4c). As previous studies have highlighted differential cell-type-specific expression of HDAC1 in GFAP-positive glia cells and HDAC2 in post-mitotic neurons of the murine brain (MacDonald and Roskams 2008; Hagelkruys et al. 2014, and Supplementary Fig. 2a–d), we next surveyed the expression of Hdac1 in the killifish brain. Due to the fact that the *N. furzeri* genome lacks an *hdac2* copy, we hypothesized to detect killifish Hdac1 in glia and neurons concomitantly. Indeed, co-localization analysis of DAPI- and Hdac1-positive nuclei in the killifish brain showed that the vast majority of cells (Spearman’s co-efficient *R* = 0.6955) were double-labeled with the exceptions of erythrocyte and endothelia cell nuclei (Supplementary Fig. 2e–g).

The staining pattern was very similar for all Hdacs tested, yet a significant difference exists in subcellular localization: Hdac1 and Hdac3 staining was strictly nuclear, whereas Hdac8 displayed cytoplasmic staining, which was particularly prominent in hypothalamic regions. The latter signal was strictly peri-nuclear (Fig. 4e), whereas, in other regions (e.g., the optic tectum), signal was present over different cytoplasmic areas. These results agree with the previous findings that Hdac8 shows cytoplasmic localization in the xenopus brain and human normal smooth muscle tissues (Waltregny et al. 2005; Guo et al. 2015). A differential expression pattern for class I Hdacs was found in the liver. Both Hdac1 and 3 were robustly expressed, whereas Hdac8 was not detectable by IHC in this organ (Fig. 4d). A similar expression pattern could be observed for gut tube and associated organs. Prominent Hdac1 and Hdac3 signal was observed in gut epithelium and in pancreatic tissue. Similar to liver, Hdac8 expression was absent in these organs (Fig. 4d). In skeletal muscles, both Hdac1 and Hdac3 expression could be observed, although Hdac3 staining was consistently lower than the signal for Hdac1 and both enzymes presented a strictly nuclear localization (Fig. 4f). Consistent with western blotting results (Fig. 4b), Hdac8 expression was high in skeletal muscle tissue and the signal localization was extranuclear (Supplementary Fig. 1a). In oblique sections, the cytoplasmic signal displayed a striated pattern resembling costamere staining, a striated pattern resulting from desmin arrangement connecting the Z-discs of the sarcomeres to the sarcolemma. To probe if this particular staining pattern arises due to Hdac8 antibody cross reactions with cytoskeletal proteins, we subjected the epitope of our Hdac8 antibody to a BLAST search, but found no accordance to known cytoskeletal proteins (data not shown). To test whether the strong Hdac8 signal in skeletal muscle could be verified by equivalent *hdac8* mRNA expression, we performed quantitative Real-time (qRT) PCR comparing *hdac8* mRNA levels in muscle, liver, and brain (Supplementary Fig. 1b). We found *hdac8* mRNA in muscle to be lower than brain, suggesting the possibility that the Hdac8 antibody recognizes an unspecific protein component in killifish striated muscle and, therefore, excluded Hdac8 antibody-dependent experiments from our consecutive analyses. Collectively, our data show that the brain is the organ with highest expression levels of all three enzymes tested. In general, Hdacs 1 and 3 show an overlapping expression pattern, whereas Hdac8 displays a differential localization both at the level of organs and with respect to subcellular, i.e., cytoplasmic localization.

### Down-regulation of Hdac1 in brain, liver, and muscle with age

**Fig. 5:**
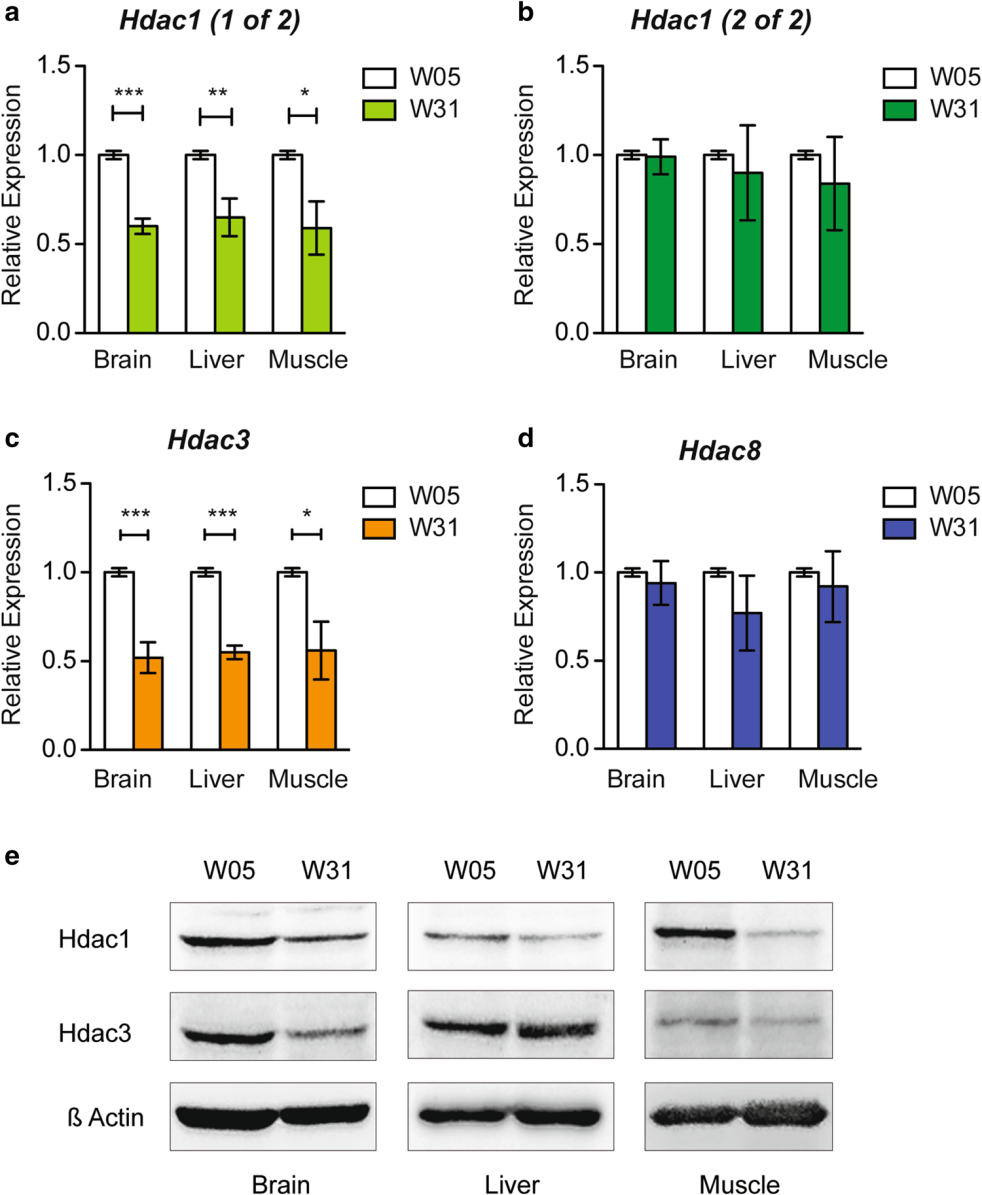
Age-dependent down-regulation of *hdac1* in brain, liver, and muscle in *N. furzeri*. Real-time PCR of **a** *hdac1* copy 1 of 2, **b** *hdac1* copy 2 of 2, **c** *hdac3* and **d** *hdac8* in *N. furzeri* brain, liver, and muscle tissue cDNA samples at 5 (W05) and 31 (W31) weeks of age in triplicate. Class I *hdac* expression is normalized to TATA-binding protein (*tbp*) housekeeping gene expression. All W05 data points are set to 1. All error bars represent ± SD. Students unpaired *t* test: ns *p* > 0.05; *p < 0.05; **p < 0.01; ***p < 0.001. **e** Western blots of brain, liver, and muscle *N. furzeri* protein extracts at 5 (W05) and 31 (W31) weeks of age. Membranes were probed with antibodies against Hdac1, Hdac3, and β-Actin as a loading control

Experiments from whole killifish protein extracts indicated divergent expression levels of class I Hdacs during aging (Fig. 3b). To monitor tissue-specific expression levels of class I Hdacs during the aging process, we performed qRT PCR and western blot analyses. We compared young (W05) and old (W31) *N. furzeri* brain, liver, and muscle tissues for expression of both copies of *hdac1, hdac3*, and *hdac8* (Fig. 5). We detected a significant age-dependent down-regulation of *hdac1* (copy 1 of 2) in all tested tissues (Fig. 5a), whereas the second copy of *hdac1* (copy 2 of 2) did not show reduced transcript levels with age (Fig. 5b). In addition, *hdac3* was significantly decreased in brain, liver, and muscle at 31 weeks of age (Fig. 5c). *Hdac8*, which has been suggested to be involved in skull morphogenesis in mammals (Haberland et al. 2009), did not show a significant down-regulation in *N. furzeri* tissues upon aging (Fig. 5d). To validate the significance of qRT PCR data on the protein level, we performed western blot analyses of Hdac1 and Hdac3 in brain, liver, and muscle extracts in both age groups (Fig. 5e). We focused on Hdac1 and Hdac3, because these class I Hdacs were significantly down-regulated on the transcriptional level (Fig. 5a, c). In the case of Hdac1, we found robust down-regulation of the protein in all tissues with age. It is, however, worth mentioning that the Hdac1 antibody does not distinguish between the two RNA products of the genetic *hdac1* duplication in the *N. furzeri* genome, and this results in a combinatorial readout of the two *hdac1* mRNA variants on the protein level. We, therefore, hypothesize that *hdac1* copy 1 of 2 mainly contributes to the decreasing protein levels during aging. The same down-regulation was observed for Hdac3 in brain and muscle, whereas, in liver, we did not detect a clear protein reduction upon aging.

### Age-dependent down-regulation of Hdac1 is accompanied by up-regulation of p21

HDAC1 has been identified as a direct negative regulator of cyclin-dependent kinase inhibitors (CDKI) such as p21 to promote unrestricted cell proliferation and cell cycle override in human tumor cells (Lagger et al. 2003) and mouse ES cells (Lagger et al. 2002; Zupkovitz et al. 2010). In mouse embryos, genetic deletion of *Hdac1* led to severe phenotypic consequences, accompanied by up-regulation of p21 and reduced proliferation rates (Lagger et al. 2002). Chemical HDAC inhibition by HDAC inhibitors (HDACi) caused comparable effects in various cell types (Glozak and Seto 2007). In summary, one of the critical functions of HDAC1 is cell cycle control via negative regulation of the CDKI *p21*.

**Fig. 6:**
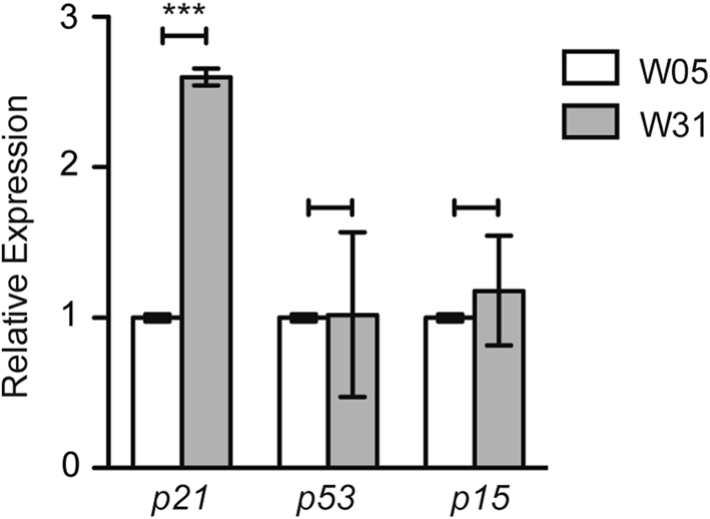
*Hdac1* down-regulation in brain correlates with increased expression of cyclin-dependent kinase (CDK) inhibitor *p21*. Real-time PCR of CDK inhibitors *p21, p53* and *p15* in *N. furzeri* whole fish cDNA extracts at 5 (W05) and 31 (W31) weeks of age in triplicate. CDK inhibitor expression is normalized to TATA-binding protein (*tbp*) housekeeping gene expression. All W05 data points are set to 1. All error bars represent ± SD. Students unpaired *t* test: ns *p* > 0.05; *p < 0.05; **p < 0.01; ***p < 0.001

As we detected robust down-regulation of Hdac1 in the brain, liver, and muscle of old fish (Fig. 5e), we next asked whether this change is accompanied by fluctuations in the expression of *N. furzeri* homologues of mammalian CDKI *p21, p16*, and *p53* exemplified in the brain. To answer this question, we compared the mRNA levels of fish *cdkn1a, cdkn2a*/*b*, and *tp53* in the brain of W05–W31 old fish using qRT PCR (Fig. 6). We detected a more than 2.5-fold increase in *p21* mRNA, whereas *p53* and *p16* levels remained unchanged. These results suggest a p53 independent activation of p21 during the aging process of *N. furzeri*.

### Down-regulation of class I HDAC expression with age in the mouse brain

**Fig. 7:**
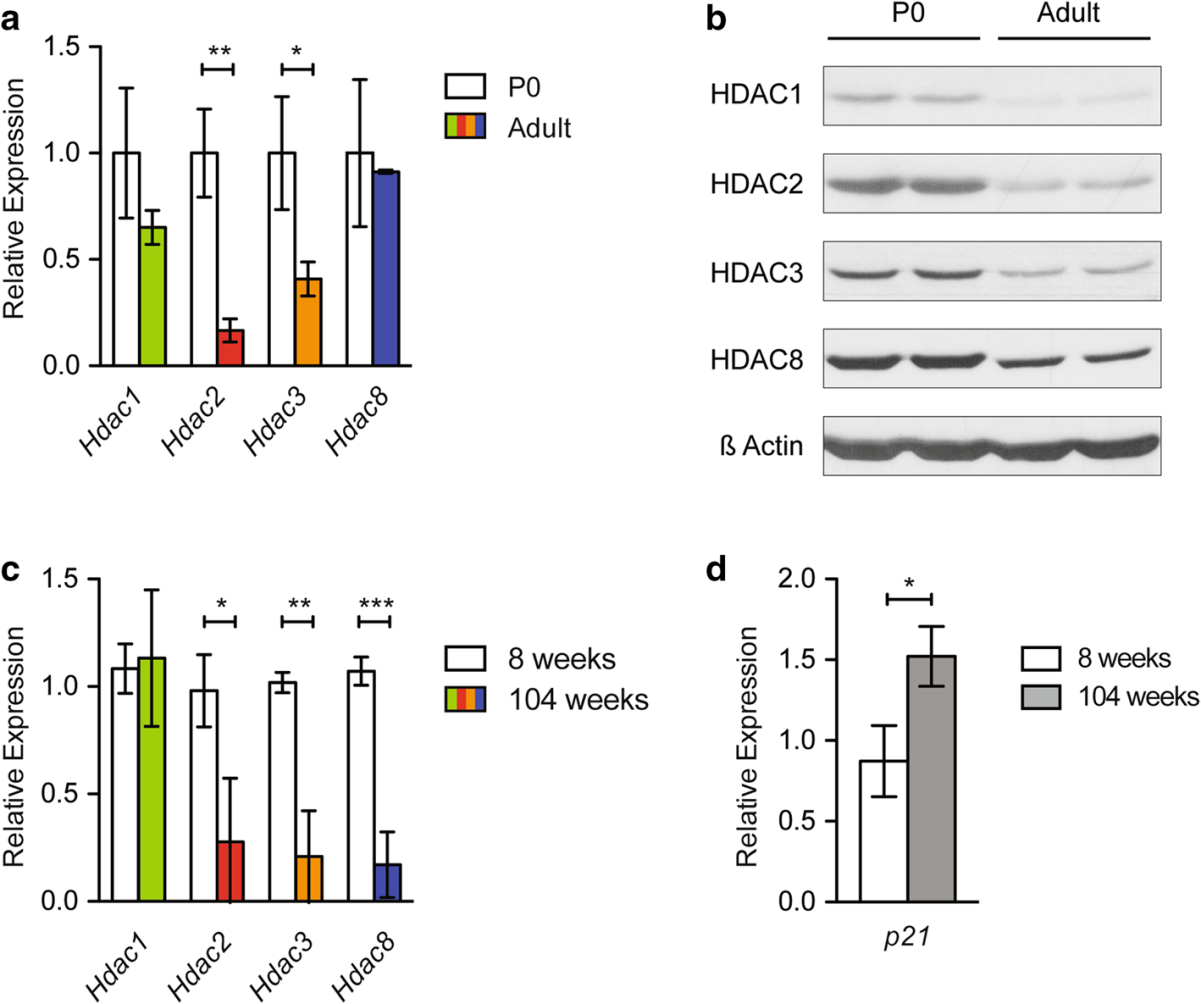
Analogous down-regulation of class I HDACs in the mammalian brain. **a** Real-time PCR of class I *Hdacs Hdac1, Hdac2, Hdac3*, and *Hdac8* cDNA samples in newborn (P0) and adult whole mouse brain in triplicate. P0 data points were set to 1. **b** Western blot of class I HDACs HDAC1, HDAC2, HDAC3, and HDAC8 in newborn (P0) and adult whole mouse brain extracts in duplicate. β-Actin was used as a loading control. **c** Real-time PCR of class I *Hdacs Hdac1, Hdac2, Hdac3*, and *Hdac8* cDNA samples in 8 and 104 weeks old whole mouse brain in triplicate. 8 weeks old data points were set to 1. **d** Real-time PCR of cyclin-dependent kinase (CDK) inhibitor *p21* cDNA samples in 8 and 104 weeks old whole mouse brain in triplicate. All mouse real-time PCR data were normalized to the housekeeping gene *Cyclophilin A*. All error bars represent ± SD. Students unpaired *t* test: ns *p* > 0.05; *p < 0.05; **p < 0.01; ***p < 0.001

To assess potential similarities or discrepancies in class I HDAC expression between species upon aging, we next compared the expression patterns of young and old N. *furzeri* brains (Fig. 5) to mouse brains of varying age groups (Fig. 7). We detected mild down-regulation of *Hdac1*, and a significant decrease in *Hdac2* and *Hdac3* levels when we compared newborn (P0) to adult (8 weeks old) mouse brains by qRT PCR (Fig. 7a). In contrast, *Hdac8* mRNA did not reveal a significant reduction during postnatal development (Fig. 7a). These findings agree well with the results obtained from our comparable studies in killifish brains (Fig. 5a–d). Interestingly, we found down-regulation of all murine brain class I HDACs on the protein level, indicating differences in protein or mRNA stability among individual class I members (Fig. 7b). To analyze the effect of aging on HDAC expression in the mouse system, we performed qRT PCR on adult (8 weeks old) and old (104 weeks old) mouse brains. Apart from *Hdac1*, all other class I members revealed decreased expression on the RNA level (Fig. 7c), accompanied by increased expression of the CDKI *p21* (Fig. 7d). These results hint towards a conserved mechanism of HDAC down-regulation between fish and mammals in the aging brain. Similarly, p21 seems to be responsive to HDAC levels in both species, enforcing its crucial function as an age-regulated gene.

## Discussion

### Evolutionary relationships of class I Hdacs

In this study, we identified class I Hdacs and analyzed their expression patterns in the annual teleost fish *N. furzeri*, an emerging novel model system for aging studies.

Database search of recently published *N. furzeri* genomes (Reichwald et al. 2015; Valenzano et al. 2015) revealed four distinct genes: two copies of *hdac1* and one copy of each *hdac3* and *hdac8*. We did not identify an *hdac2* gene in the *N. furzeri* genome, which distinguishes killifish from its closest relatives by encoding a unique *hdac1* duplication and *hdac2* absence of copy number variation (2-X-1-1) (Fig. 1). It has previously been suggested that these two genes originate from an early duplication of a vertebral common ancestral *hdac1*/*2* gene (Gregoretti et al. 2004). It is, however, unlikely that one of the two *hdac1* copies found in teleost fish later emerged as *hdac2* gene due to several reasons: first, most teleost fish harbor an *hdac2* gene in addition to their two copies of *hdac1* (Fig. 1), and second, *N. furzeri* shares genetic microsynteny of its *hdac1* genes with other species. Interestingly, some of the neighboring genes of both copies of teleost *hdac1* are located on the same chromosome in relative vicinity to the human *HDAC1* gene. In contrast, we did not detect *N. furzeri hdac1* surrounding genes in vicinity to the *HDAC2* gene in any other species. Therefore, the gene duplication of a common ancestral *hdac1*/*2* gene must have occurred significantly earlier than the suggested teleost-specific gene amplification.

*Hdac3*, which defines its own subgroup of class I Hdacs, was present in all analyzed species, similarly showing high conservation between species. Interestingly, *hdac8* representing the most distinct member of this group was found in all species analyzed except for the early diverged lamprey.

### Expression patterns of class I HDACs during development

We have next addressed expression patterns of class I Hdacs in the developing *N. furzeri* embryo (Fig. 2), and their presence and localization in adult neuronal, metabolic, and locomotor tissues such as brain, liver, and muscle (Figs. 3, 4, 5). The spatio-temporal expression patterns of class I Hdacs throughout the lifespan of killifish change from ubiquitous in black eye-stage embryos towards tissue-specific patterns in adults. Some class I Hdacs become up- or down-regulated in certain tissues such as the gut tube. Importantly, however, all class I Hdacs are highly expressed in brain neuronal cells in areas known to be proliferative in *N. furzeri* (Tozzini et al. 2012) and zebrafish (Kizil et al. 2012). For example, in midbrain, particularly strong signal was detected in the caudal periventricular grey zone (PGZ) region, known to supply the optic tectum with new neuronal cells in zebrafish (Ito et al. 2010). The strong signal in the optic tectum may also be connected to the life-long growth of the eyes of *N. furzeri*. Indeed, old fish have larger eyes than younger ones and this growth requires the constant and strict adaptation of the retinotopic projections and correlates with continuous growth of the optic tectum (Cerveny et al. 2012).

Considering the high conservation of these enzymes amongst species, it is not surprising that class I Hdac expression patterns in *N. furzeri* resemble the HDAC expression profile of other vertebrates. For example, class I HDACs are highly expressed in the developing mouse and chicken brain (Murko et al. 2010) and in the zebrafish central nervous system (Yamaguchi et al. 2005). The adult mouse brain represents an interesting deviation from well-described HDAC1 and HDAC2 expression patterns in various proliferating cell types. HDAC1 is expressed in neural stem cells and mature glia, while HDAC2 is detected in neural progenitors and post-mitotic neurons (MacDonald and Roskams 2008; Hagelkruys et al. 2014, and Supplementary Fig. 2). HDAC2 has additionally been identified as an essential regulator of neuronal differentiation, because a single allele of *Hdac2* but not *Hdac1* was sufficient to ensure normal brain development in the absence of its paralog (Hagelkruys et al. 2014). In contrast, the sole Hdac1 copy in zebrafish is expressed from the early embryogenesis onwards and is indispensable for differentiation of retina cells into neurons (Yamaguchi et al. 2005). Because *N. furzeri* similarly to zebrafish lacks the *hdac2* gene, the absence of Hdac2 in the brain is most likely compensated by either one or both of the two copies of Hdac1. Further studies using isotype-specific antibodies are required to determine differential expression patterns and tissue-specific functions of both Hdac1 isoforms.

Hdac8 reveals the most prominent differences in cellular localization and tissue expression when compared to other class I members. Unlike Hdac1 and Hdac3 that are localized to the nucleus and expressed in all analyzed tissues, Hdac8 shows peri-nuclear to cytoplasmic localization and no presence in liver of adult fish (Fig. 4d, e). Similar to our findings in *N. furzeri*, northern blot analysis of human multiple tissue blots detected highest levels of *HDAC8* mRNA in brain but significantly lower levels in liver. Moreover, the same study finds mainly peri-nuclear localization of HDAC8 in brain (Van den Wyngaert et al. 2000). Another study shows that HDAC8 is mainly expressed in mammalian smooth muscle cells accompanied by prominent cytoplasmic localization (Waltregny et al. 2005).

### Class I HDACs during the aging process

The role of HDACs in the regulation of aging has been controversial, as several studies produced conflicting results regarding in- or decreased cellular acetylation levels upon age in different tissues in various model organisms.

To investigate individual class I Hdacs as a function of age, we surveyed the expression of *N. furzeri* Hdacs in three functionally different tissues: brain, liver, and muscle. Upon aging, the levels of Hdac1 were decreased in whole fish extracts and in all three tissues investigated (Figs. 3b, 5). Similar to our findings, it was shown that the amount of *rpd3* mRNA, a class I Hdac homologue in budding yeast, decreased with age, whereas deletion of *rpd3* resulted in prolonged lifespan (Kim et al. 1999). Hdac3 remained unchanged in whole fish extracts, but was differentially expressed in individual tissues (Figs. 3b, 5). We detected an age-dependent down-regulation of Hdac3 in brain and muscle, whereas Hdac3 levels in liver remained constant. This raises the question whether, in liver, Hdac3 exhibits increased protein stability as it has been suggested that its absence leads to hepatocellular carcinoma and disrupted lipid and cholesterol homeostasis in mice (Knutson et al. 2008). Our findings are in accordance with a recent study characterizing whole-genome transcript regulation during brain aging of *N. furzeri* MZM 0410, the exact same strain used in this study. Transcriptomic profiling covering the entire adult killifish life cycle also showed down-regulation of *Hdac1* and *Hdac3* in the aging brain (Baumgart et al. 2014). As age-dependent down-regulation of class I HDACs is most prominently detected in brain of killifish, we compared our findings to the mouse as an independent vertebrate system. Indeed, total mouse brain extracts isolated from young and old animals similarly showed an age-dependent overall decrease in protein levels for all class I HDACs (Fig. 7b), whereas mRNA levels seemed more dynamically regulated (Fig. 7a). Upon aging, we detected a clear down-regulation of class I *Hdacs* with the exception of *Hdac1* (Fig. 7c). Age-dependent down-regulation of HDAC2 but not HDAC1 in mouse brain could be explained by the cell-type-specific expression pattern of these enzymes (MacDonald and Roskams 2008; Hagelkruys et al. 2014). HDAC1 as a regulator of cellular proliferation might be essential to ensure glia cell propagation and differentiation, whereas HDAC2 could overtake divergent functions in post-mitotic neurons. Decreased levels of HDAC2 and HDAC3 were also reported in Purkinje neurons in the cerebellum of old rats (Khurana and Dlugos 2017). Similar to these findings, class I HDACs in oligodendrocytes of the mouse corpus callosum displayed an age-dependent decrease in expression (Shen et al. 2008). However, another study reported beneficial effects of HDAC2 knockout on memory formation and synaptic plasticity, once again emphasizing the feasibility of HDACi treatment in brain malignancies (Guan et al. 2009). In summary, these findings suggest distinct age-related functions of class I HDACs that depend on physiological cell-type requirements, brain region, and age of the animal.

Finally, we have also demonstrated that decreased expression of class I HDACs in brains of old killifish and mice is accompanied with increased transcription of the *Cdkn1a p21* gene (Figs. 6, 7d). This finding is consistent with a previous study reporting transcriptional up-regulation of *p21* during the aging process in brain of *N. furzeri* (Baumgart et al. 2014). Up-regulation of one or several cyclin-dependent kinase inhibitors and tumor suppressor genes such as *p16Ink4a, p15Ink4b, Cdkn1a p21, p53*, and the mitogen-activated protein kinase *p38MAPK* are often associated with cellular senescence. For example, DNA damage in Purkinje and cortical neurons led to activation of *p21, p38MAPK* and elevated β-gal staining (Jurk et al. 2012). Interestingly, we did not detect an age-dependent change in expression levels of the *p53* gene in brains of killifish (Fig. 6). Similar to our findings in *N. furzeri* brains, skin isolated from old fish displayed elevated expression levels of *p21* but no change in *p53* (Graf et al. 2013). The tumor suppressor gene p53 is described as one of the key regulators of *p21* transcription by binding to specific binding sites on the *p21* promoter (El-Deiry et al. 1993). In addition, HDAC1 and p53 act as antagonists by competing for the interaction with the SP1 transcription factor that is necessary for *p21* expression in mammalian cells (Lagger et al. 2003).

Furthermore, p53 was one of the first non-histone proteins shown to be acetylated (Gu and Roeder 1997). The acetylation of p53 is associated with protein stabilization, activation, and stimulation of its specific DNA binding (Brooks and Gu 2011). Conversely, it was demonstrated that deacetylation of p53 mediated via an HDAC1-containing complex strongly represses p53-dependent transcriptional activation of *p21* (Luo et al. 2000). Therefore, the reduction of HDAC1 can affect the repressor/activator balance on the *p21* promoter and the acetylation status of p53 protein, leading to increased expression of *p21* without an actual change in *p53* levels. In addition, it has been demonstrated that HDACi, such as trichostatin A (TSA) and MS275 can positively affect the expression of the *p21* gene (Lagger et al. 2003; Zupkovitz et al. 2006; Jurkin et al. 2011).

In summary, our results demonstrate that aging, at least in neural, metabolic, and locomotor tissues, is accompanied with decreased levels of class I HDACs that result in cell cycle arrest and senescence-like phenotypes via induction of *p21*. Moreover, high-sequence identity and similar age-dependent expression of class I HDACs in different vertebrate species suggest their involvement, in general, evolutionary conserved pathways affecting the regulation of age-related degeneration and diseases. In the last few decades, vast knowledge about class I HDACs has been gained through the use of HDACi. Although most of these inhibitors lack specificity towards single HDACs, they showed certain beneficial effects in the treatment of tumor cells leading to apoptosis, cell cycle arrest, and differentiation, implying that these agents might additionally have effects on the aging process. Therefore, further studies involving the use of HDACi and creation of transgenic fish will help to better understand the role of specific HDAC members and their mode of regulation during the aging process.

## Electronic supplementary material

Below is the link to the electronic supplementary material.

Supplementary material 1 (DOCX 10937 KB)

## References

[CR1] Amemiya CT, Alföldi J, Lee AP (2013). The African coelacanth genome provides insights into tetrapod evolution. Nature.

[CR2] Baumgart M, Groth M, Priebe S (2014). RNA-seq of the aging brain in the short-lived fish N. furzeri—conserved pathways and novel genes associated with neurogenesis. Aging Cell.

[CR3] Braasch I, Gehrke AR, Smith JJ (2016). The spotted gar genome illuminates vertebrate evolution and facilitates human-teleost comparisons. Nat Genet.

[CR4] Brooks CL, Gu W (2011). The impact of acetylation and deacetylation on the p53 pathway. Protein Cell.

[CR5] Brunmeir R, Lagger S, Seiser C (2009). Histone deacetylase HDAC1/HDAC2-controlled embryonic development and cell differentiation. Int J Dev Biol.

[CR6] Cerveny KL, Varga M, Wilson SW (2012). Continued growth and circuit building in the anamniote visual system. Dev Neurobiol.

[CR7] Chang KT, Min K-T (2002). Regulation of lifespan by histone deacetylase. Ageing Res Rev.

[CR8] El-Deiry WS, Tokino T, Velculescu VE (1993). WAF1, a potential mediator of p53 tumor suppression. Cell.

[CR9] Genade T, Benedetti M, Terzibasi E (2005). Annual fishes of the genus Nothobarnchius as a model system for aging research. Aging Cell.

[CR10] Glozak MA, Seto E (2007). Histone deacetylases and cancer. Oncogene.

[CR11] Graf M, Cellerino A, Englert C (2010). Gender separation increases somatic growth in females but does not affect lifespan in *Nothobranchius furzeri*. PLoS One.

[CR12] Graf M, Hartmann N, Reichwald K, Englert C (2013). Absence of replicative senescence in cultured cells from the short-lived killifish *Nothobranchius furzeri*. Exp Gerontol.

[CR13] Gregoretti I, Lee Y-M, Goodson HV (2004). Molecular evolution of the histone deacetylase family: functional implications of phylogenetic analysis. J Mol Biol.

[CR14] Gu W, Roeder RG (1997). Activation of p53 sequence-specific DNA binding by acetylation of the p53 C-terminal domain. Cell.

[CR15] Guan J-S, Haggarty SJ, Giacometti E (2009). HDAC2 negatively regulates memory formation and synaptic plasticity. Nature.

[CR16] Guo X, Ruan H, Li X (2015). Subcellular localization of class I histone deacetylases in the developing xenopus tectum. Front Cell Neurosci.

[CR17] Haberland M, Mokalled MH, Montgomery RL, Olson EN (2009). Epigenetic control of skull morphogenesis by histone deacetylase 8. Genes Dev.

[CR18] Hagelkruys A, Lagger S, Krahmer J (2014). A single allele of Hdac2 but not Hdac1 is sufficient for normal mouse brain development in the absence of its paralog. Development.

[CR19] Hartmann N, Reichwald K, Lechel A (2009). Telomeres shorten while Tert expression increases during ageing of the short-lived fish *Nothobranchius furzeri*. Mech Ageing Dev.

[CR20] Hoppe B, Pietsch S, Franke M (2015). MiR-21 is required for efficient kidney regeneration in fish. BMC Dev Biol.

[CR21] Ito Y, Tanaka H, Okamoto H, Ohshima T (2010). Characterization of neural stem cells and their progeny in the adult zebrafish optic tectum. Dev Biol.

[CR22] Jurk D, Wang C, Miwa S (2012). Postmitotic neurons develop a p21-dependent senescence-like phenotype driven by a DNA damage response. Aging Cell.

[CR23] Jurkin J, Zupkovitz G, Lagger S (2011). Distinct and redundant functions of histone deacetylases HDAC1 and HDAC2 in proliferation and tumorigenesis. Cell Cycle.

[CR24] Kaeberlein M, McVey M, Guarente L (1999). The SIR2/3/4 complex and SIR2 alone promote longevity in Saccharomyces cerevisiae by two different mechanisms. Genes Dev.

[CR25] Kang H-L, Benzer S, Min K-T (2002). Life extension in Drosophila by feeding a drug. Proc Natl Acad Sci.

[CR26] Khurana A, Dlugos CA (2017). Age-related alterations in histone deacetylase expression in Purkinje neurons of ethanol-fed rats. Brain Res.

[CR27] Kim S, Benguria A, Lai CY, Jazwinski SM (1999). Modulation of lifespan by histone deacetylase genes in *Saccharomyces cerevisiae*. Mol Biol Cell.

[CR28] Kim Y, Nam HG, Valenzano DR (2016). The short-lived African turquoise killifish: an emerging experimental model for ageing. Dis Model Mech.

[CR29] Kizil C, Kaslin J, Kroehne V, Brand M (2012). Adult neurogenesis and brain regeneration in zebrafish. Dev Neurobiol.

[CR30] Knutson SK, Chyla BJ, Amann JM (2008). Liver-specific deletion of histone deacetylase 3 disrupts metabolic transcriptional networks. EMBO J.

[CR31] Lagger G, O’Carroll D, Rembold M (2002). Essential function of histone deacetylase 1 in proliferation control and CDK inhibitor repression. EMBO J.

[CR32] Lagger G, Doetzlhofer A, Schuettengruber B (2003). The tumor suppressor p53 and histone deacetylase 1 are antagonistic regulators of the cyclin-dependent kinase inhibitor p21/WAF1/CIP1 Gene. Mol Cell Biol.

[CR33] Luo J, Su F, Chen D (2000). Deacetylation of p53 modulates its effect on cell growth and apoptosis. Nature.

[CR34] MacDonald JL, Roskams AJ (2008). Histone deacetylases 1 and 2 are expressed at distinct stages of neuro-glial development. Dev Dyn.

[CR35] Montgomery RL, Davis CA, Potthoff MJ (2007). Histone deacetylases 1 and 2 redundantly regulate cardiac morphogenesis, growth, and contractility. Genes Dev.

[CR36] Montgomery RL, Potthoff MJ, Haberland M (2008). Maintenance of cardiac energy metabolism by histone deacetylase 3 in mice. J Clin Invest.

[CR37] Murko C, Lagger S, Steiner M (2010). Expression of class I histone deacetylases during chick and mouse development. Int J Dev Biol.

[CR38] Murko C, Lagger S, Steiner M (2013). Histone deacetylase inhibitor Trichostatin A induces neural tube defects and promotes neural crest specification in the chicken neural tube. Differentiation.

[CR39] Pasyukova EG, Vaiserman AM (2017). HDAC inhibitors: A new promising drug class in anti-aging research. Mech Ageing Dev.

[CR40] Petzold A, Reichwald K, Groth M (2013). The transcript catalogue of the short-lived fish *Nothobranchius furzeri* provides insights into age-dependent changes of mRNA levels. BMC Genom.

[CR41] Reichwald K, Petzold A, Koch P (2015). Insights into sex chromosome evolution and aging from the genome of a short-lived fish. Cell.

[CR42] Rogina B, Helfand SL, Frankel S (2002). Longevity regulation by Drosophila Rpd3 deacetylase and caloric restriction. Science.

[CR43] Shen S, Liu A, Li J (2008). Epigenetic memory loss in aging oligodendrocytes in the corpus callosum. Neurobiol Aging.

[CR44] Terzibasi E, Lefrançois C, Domenici P (2009). Effects of dietary restriction on mortality and age-related phenotypes in the short-lived fish *Nothobranchius furzeri*. Aging Cell.

[CR45] Tozzini ET, Baumgart M, Battistoni G, Cellerino A (2012). Adult neurogenesis in the short-lived teleost *Nothobranchius furzeri*: localization of neurogenic niches, molecular characterization and effects of aging. Aging Cell.

[CR46] Trivedi CM, Luo Y, Yin Z (2007). Hdac2 regulates the cardiac hypertrophic response by modulating Gsk3β activity. Nat Med.

[CR47] Valenzano DR, Cellerino A (2006). Resveratrol and the pharmacology of aging: a new vertebrate model to validate an old molecule. Cell Cycle.

[CR48] Valenzano DR, Terzibasi E, Cattaneo A (2006). Temperature affects longevity and age-related locomotor and cognitive decay in the short-lived fish: *Nothobranchius furzeri*. Aging Cell.

[CR49] Valenzano DR, Benayoun BA, Singh PP (2015). The African turquoise killifish genome provides insights into evolution and genetic architecture of lifespan. Cell.

[CR50] Van den Wyngaert I, de Vries W, Kremer A (2000). Cloning and characterization of human histone deacetylase 8. FEBS Lett.

[CR51] Waltregny D, Glénisson W, Tran SL (2005). Histone deacetylase HDAC8 associates with smooth muscle -actin and is essential for smooth muscle cell contractility. FASEB J.

[CR52] Wendler S, Hartmann N, Hoppe B, Englert C (2015). Age-dependent decline in fin regenerative capacity in the short-lived fish *Nothobranchius furzeri*. Aging Cell.

[CR53] Yamaguchi M, Tonou-Fujimori N, Komori A (2005). Histone deacetylase 1 regulates retinal neurogenesis in zebrafish by suppressing Wnt and Notch signaling pathways. Development.

[CR54] Zupkovitz G, Tischler J, Posch M (2006). Negative and positive regulation of gene expression by mouse histone deacetylase 1. Mol Cell Biol.

[CR55] Zupkovitz G, Grausenburger R, Brunmeir R (2010). The cyclin-dependent kinase inhibitor p21 is a crucial target for histone deacetylase 1 as a regulator of cellular proliferation. Mol Cell Biol.

