# Supplementary material for: Histone deacetylase 1 expression is inversely correlated with age in the short-lived fish Nothobranchius furzeri
Source: Histochem Cell Biol. 2018 Jun 28;150(3):255–69. doi: 10.1007/s00418-018-1687-4 (PMC6096771; doi:10.1007/s00418-018-1687-4)
Supplement: Supplementary file 1 — Supplementary material 1 (DOCX 10937 KB) [file 418_2018_1687_MOESM1_ESM.docx]

**Supporting information**

**Supplementary Figure 1**

**
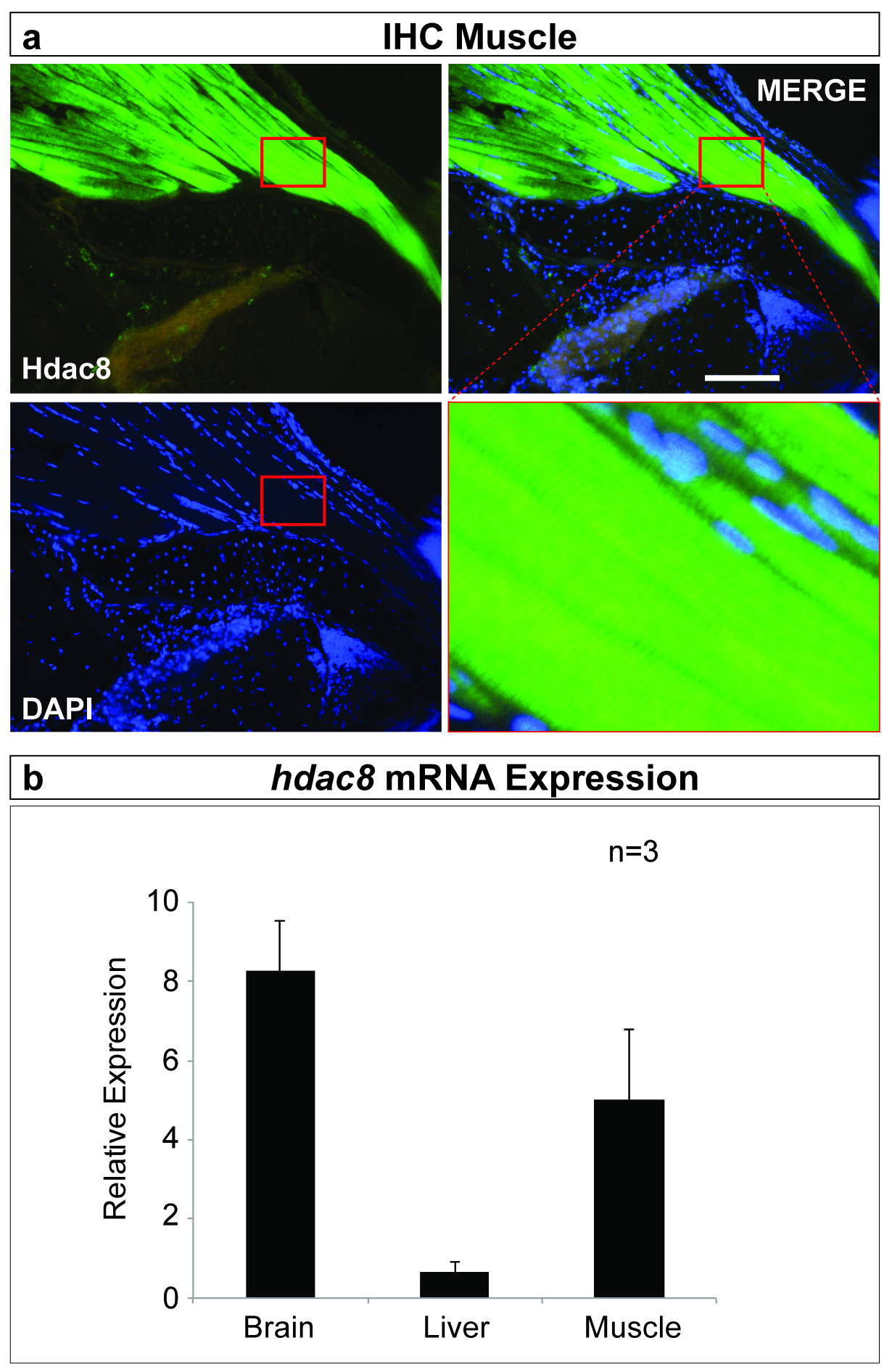
**

**Supplementary Fig.1** Hdac8 expression in skeletal muscle **a** Possible Hdac8 antibody cross reaction with cytoskeletal proteins. Fluorescent IHC stainings of Hdac8 striated muscle of 5 weeks old fish. Nuclei were counterstained with DAPI. Scale bar is 50µm. Red rectangle shows the zoomed region of muscle tissue presented in lower right panel. Scale bar: 50 μm. **b** Real Time PCR of *hdac8* in *N. furzeri* comparing brain, liver and muscle tissue cDNA samples at weeks 5 of age in triplicate. *Hdac8* expression is normalized to TATA binding protein (*tbp*) housekeeping gene expression.

**Supplementary Figure 2**

**
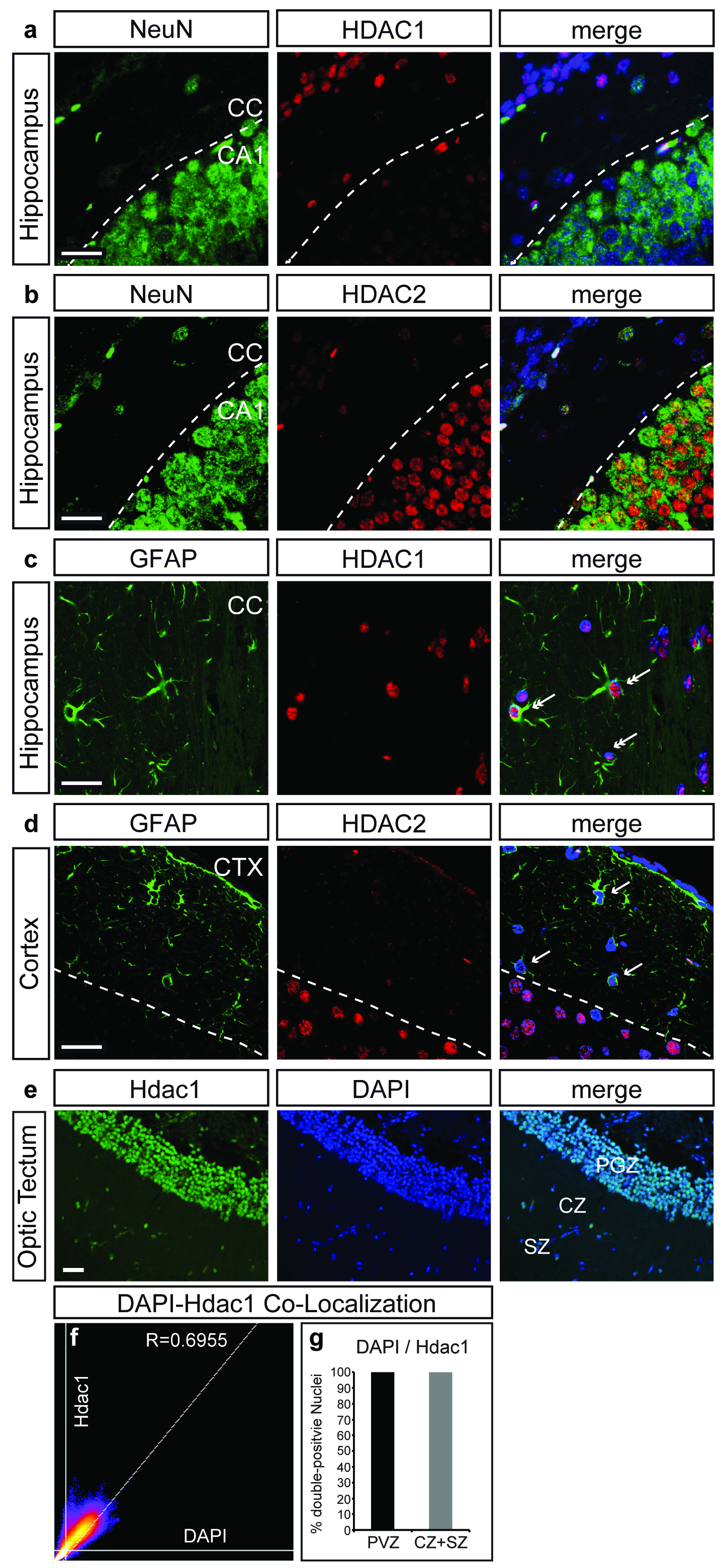
**

**Supplementary Fig.2** Cell type specific protein expression of HDAC1/HDAC2 in the murine and killifish brain **a-d** HDAC1 and HDAC2 display divergent expression patterns in the postnatal mouse brain. Fluorescence immunohistochemistry stainings of paraffin embedded brain sections of adult 129SV/BI6 mice with antibodies against HDAC1 and HDAC2 in combination with GFAP or NeuN. Nuclei are counterstained with 4’,6-diamidino-2-phenylindole (DAPI). In the mouse brain, HDAC1, but not HDAC2, is highly expressed in glial fibrillary acidic protein (GFAP)-positive astrocytes (c, d). By contrast, HDAC2, but not HDAC1, is primarily expressed in post-mitotic neurons detected by the neuronal marker neuronal nuclei (NeuN) (a, b). White double arrows (c) indicate astrocytes overlapping with HDAC1 expression, whereas white arrows (d) represent HDAC2 negative glia cells. The white dashed line indicates the border between the corpus callosum and the CA1 region of the hippocampus (a, b) and the glia rich area from neuron dense layers in the cortex (d). *CA1* hippocampal CA1 region, *CC* corpus callosum, *CTX* cortex. Scale bar: 20 μm **e-g** Hdac1 is expressed in glia and neurons in the killifish brain. **e** Fluorescence IHC stainings of Hdac1 on brain - optic tectum region from 5 weeks old *N. furzeri*. Nuclei were counterstained with DAPI. Co-localization of DAPI and Hdac1 positive nuclei was correlated in two ways using Fiji open source software. **f** Spearman´s co-localization coefficient (R) was evaluated using the PSC co-localization plugin over the entire two-channel image. **g** Labelled nuclei were manually counted employing the Cell Counter plugin in Fiji. We found that the vast majority of cells were double-labelled with the exceptions of erythrocyte and endothelia cell nuclei. Counted double-labelled nuclei were categorized according to location in PGZ and CZ+SZ nuclei. *CZ* central zone, *PGZ* periventricular grey zone, *SZ* superficial zone. Scale bar: 20 μm.

**Supplementary Table 1**

List of gene identifiers from which the protein sequences have been retrieved:

human (*Homo sapiens)*

ENSG00000116478

ENSG00000196591

ENSG00000171720

ENSG00000147099

mouse *(Mus musculus*)

ENSMUSG00000028800

ENSMUSG00000019777

ENSMUSG00000024454

ENSMUSG00000067567

frog *(Xenopus tropicalis)*

ENSXETG00000005253

ENSXETG00000007339

ENSXETG00000002708

ENSXETG00000020527

zebrafish *(Danio rerio)*

ENSDARG00000015427

ENSDARG00000037514

ENSDARG00000003021

spotted gar *(Lepisosteus oculatus)*

ENSLOCG00000014449

ENSLOCG00000016977

ENSLOCG00000002539

ENSLOCG00000012055

medaka *(Oryzias latipes)*

ENSORLG00000017990

ENSORLG00000014155

ENSORLG00000017491

ENSORLG00000009006

ENSORLG00000009606

platyfish *(Xiphophorus maculatus)*

ENSXMAG00000005199

ENSXMAG00000000379

ENSXMAG00000018986

ENSXMAG00000000849

ENSXMAG00000014850

coelacanth *(Latimeria chalumnae)*

ENSLACG00000013376

ENSLACG00000009761

ENSLACG00000011351

lamprey*(Petromyzon marinus)*

ENSPMAG00000009083

ENSPMAG00000008768

killifish:

Nfu_g_1_016470

Nfu_g_1_014999

Nfu_g_1_012108

Nfu_g_1_009605

*Sulfolobus islandicus:*

UniProtKB - C3NML0 (C3NML0_SULIN)

**Supplementary Table 2**

Antibodies used for immunohistochemistry (IHC) and western-blot (WB) analysis.

| **Protein** | **Specificity** | **Company (cat. Number)** | **Dilution IHC** | | **Dilution WB** | |
| --- | --- | --- | --- | --- | --- | --- |
|  |  |  | **Killifish** | **Mouse** | **Killifish** | **Mouse** |
| HDAC1 | Rabbit polyclonal | Abcam (ab33278) | 1:400 | - | 1:1000 | 1:1000 |
| HDAC1 | Rabbit polyclonal | Abcam (ab7028) | - | 1:500 | - | - |
| HDAC2 | Mouse monoclonal | Millipore (3F3) | - | - | - | 1:1000 |
| HDAC2 | Rabbit polyclonal | Christin Seiser | - | 1:100 | - | - |
| HDAC3 | Rabbit polyclonal | Abcam (ab7038) | 1:3000 | - | 1:5000 | 1:5000 |
| HDAC8 | Rabbit polyclonal | Abcam (ab137474) | 1:400 | - | 1:1000 | 1:1000 |
| ß-actin | Rabbit polyclonal | CST (4967) | - | - | 1:2000 | 1:2000 |
| GFAP | Mouse monoclonal | CST (3670) | - | 1:200 | - | - |
| NeuN | Mouse monoclonal | Chemicon (MAB377) | - | 1:100 | - | - |

**Supplementary Table 3**

**
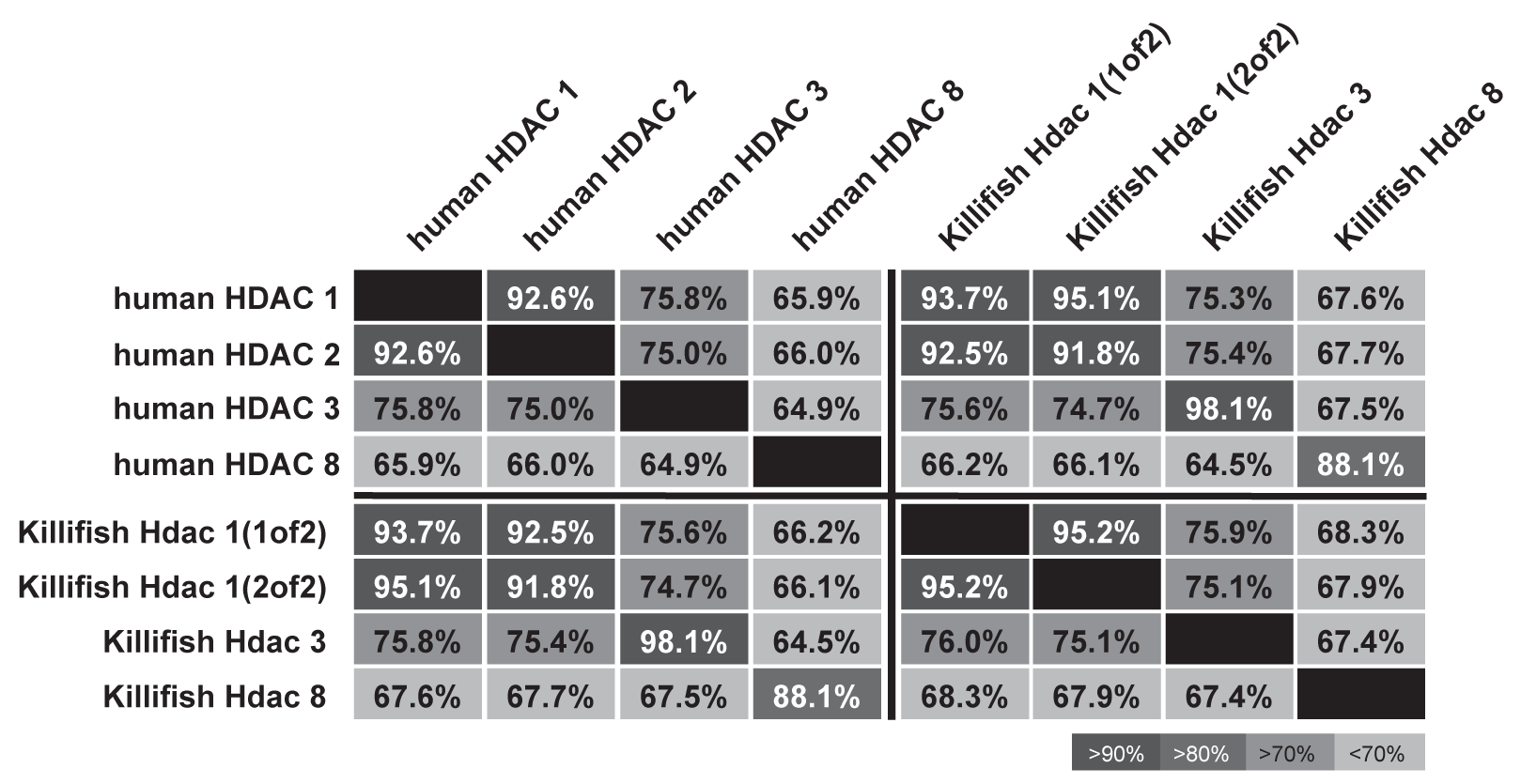
**

**Supplementary Table 3** Pairwise sequence comparison between killifish and human class I HDACs. The percentage similarity was scored using Geneious software.
